# Quantitation of deoxynucleoside triphosphates by click reactions

**DOI:** 10.1038/s41598-020-57463-3

**Published:** 2020-01-17

**Authors:** Chang-Yu Huang, Miriam Yagüe-Capilla, Dolores González-Pacanowska, Zee-Fen Chang

**Affiliations:** 1 Institute of Biochemistry and Molecular Biology, National Yang-Ming University, No.155, Sec. 2, Linong Street, Taipei, 112 Taiwan; 2 Institute of Molecular Medicine, College of Medicine, National Taiwan University, No. 1, Section 1, Jen-Ai Road, Taipei, 100 Taiwan, ROC; 3 Instituto de Parasitología y Biomedicina “López-Neyra” (IPBLN), Consejo Superior de Investigaciones Científicas. Parque Tecnológico de Ciencias de la Salud, Avenida del Conocimiento, 17, 18016 Armilla, Granada Spain; 4 Center of Precision Medicine, College of Medicine, National Taiwan University, Taipei, Taiwan

**Keywords:** Biochemical assays, Assay systems

## Abstract

The levels of the four deoxynucleoside triphosphates (dNTPs) are under strict control in the cell, as improper or imbalanced dNTP pools may lead to growth defects and oncogenesis. Upon treatment of cancer cells with therapeutic agents, changes in the canonical dNTPs levels may provide critical information for evaluating drug response and mode of action. The radioisotope-labeling enzymatic assay has been commonly used for quantitation of cellular dNTP levels. However, the disadvantage of this method is the handling of biohazard materials. Here, we described the use of click chemistry to replace radioisotope-labeling in template-dependent DNA polymerization for quantitation of the four canonical dNTPs. Specific oligomers were designed for dCTP, dTTP, dATP and dGTP measurement, and the incorporation of 5-ethynyl-dUTP or C8-alkyne-dCTP during the polymerization reaction allowed for fluorophore conjugation on immobilized oligonucleotides. The four reactions gave a linear correlation coefficient >0.99 in the range of the concentration of dNTPs present in 10^6^ cells, with little interference of cellular rNTPs. We present evidence indicating that data generated by this methodology is comparable to radioisotope-labeling data. Furthermore, the design and utilization of a robust microplate assay based on this technology evidenced the modulation of dNTPs in response to different chemotherapeutic agents in cancer cells.

## Introduction

The levels of the four dNTPs play a critical role in the maintenance of genetic stability^1^. Recent studies have demonstrated that dNTP deficiency, expansion, or imbalance due to dysregulation of nucleotide metabolism enzymes can lead to genome instability, promoting tumorigenesis^1–5^. For instance, elevation of the R2 subunit of ribonucleotide reductase (RNR) in cancer cells has been shown to expand canonical dNTP and dUTP pools, a situation that leads to increased replication stress^2,6,7^. Deficiency of cytidine deaminase causes under-replication of DNA^8,9^. Aberrant activation of oncogenes has been demonstrated to induce replication stress and DNA damage in newly transformed cells due to a decrease in dNTPs^3,5,10^. According to the KEGG database, there are, at least, 120 known enzymes implicated in nucleotide biosynthesis and more than 30 catalytic steps involved in catabolism. Modulation of nucleotide metabolism enzymes could affect cellular levels of dNTPs and also the response to therapeutic agents^11–14^. Therefore, dNTP pools can be a useful parameter for assessing mutagenesis potential and drug response. Therefore, a convenient method for quantitation of the four canonical dNTPs is in great demand.

To date, several methods have been developed to quantitate cellular levels of the four dNTPs. Among them, enzymatic- and liquid chromatography-based assays have been widely used. The latter involves the direct separation of dNTPs by HPLC which are detected by UV or coupling with tandem mass spectrometry (LC-MS/MS)^15^. The main limitations of HPLC-UV are low sensitivity, whereas LC-MS/MS methods are limited by requirements for costly instrumentation and skilled operation^15,16^. As for the enzymatic-based assay, DNA polymerases are employed to incorporate a specific dNTP in combination with another radioisotope-labeled dNTP, generally^17^ dATP, to an oligonucleotide template. The oligonucleotide template is composed of a short primer and the specificity is provided by the larger template, that is formed by TTTX repeats, where X is the dNTP to be measured. Thus, the incorporation of labeled [^3^H]dATP is proportional to the incorporation of the dNTP to be determined. For example, the amount of dCTP in a sample is calculated from the incorporated [^3^H]dATP in a standard curve of increasing amounts of dCTP. This method is an indirect measurement of dNTPs and has been commonly used. However, the disadvantage of this technique has been the handling storage and disposal of radioactive isotopes in line with government or institute regulations. To solve this issue, dual-quenched fluorophore-labeled oligonucleotides for dNTP quantification were developed^18^. The primer-dependent extension reaction in the assay detects the release of fluorescence-labeled nucleotides from the oligo-probe with quenchers annealed at the 5′end of the template via the 5′-3′exonuclease activity of Taq polymerase. The potential problem of this method is the stability of the probe containing the conjugated 6-Carboxyfluorescein (6-FAM) and the quencher during storage over long time periods. In this study, we used a copper(I)-catalyzed alkyne-azide cycloaddition (CuAAC) “click” reaction to establish a quantitation assay that overcomes the major drawbacks of the aforementioned methods. The method was adapted to a microplate assay in order to increase analysis capacity. We used this assay to demonstrate that dNTP levels are modified in response to different chemotherapeutic agents.

## Results

### The combination of enzymatic and click reactions for dNTP quantification

**Figure 1:**
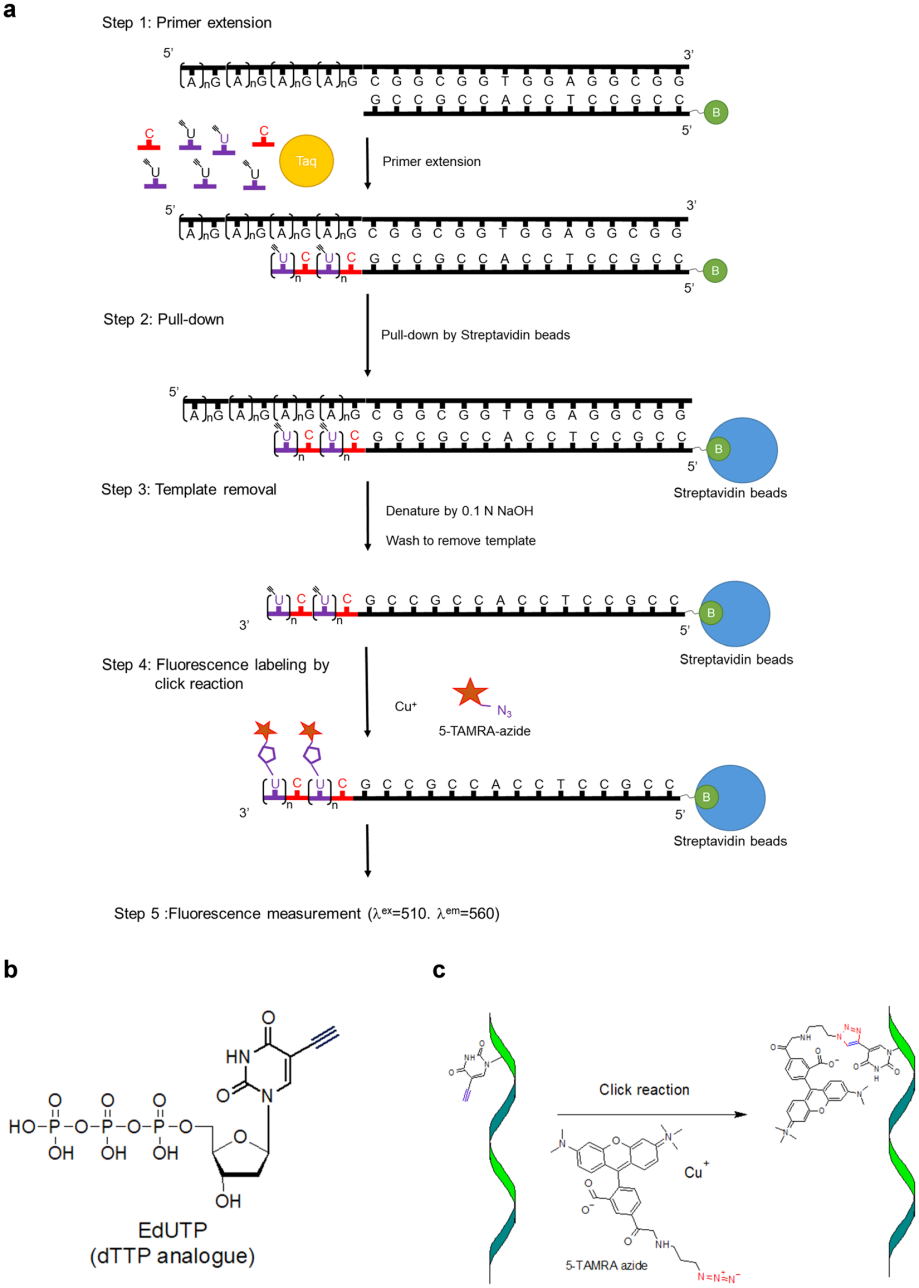
Schematic diagram of dNTP quantification by a combination of the enzymatic reaction and the click assay. (**a**) The specific template oligomer designed for dCTP measurements was annealed with the biotin-labeled primer. In the presence of dCTP, DNA polymerase extends from the 3′ end and EdUTP is incorporated. The newly synthesized DNA is pulled down using streptavidin Sepharose. After denaturation, EdU incorporated into ssDNA is labeled with the fluorophore 5-TAMRA by click reaction. The values of fluorescence units are proportional to the incorporated dCTP. (**b**) The chemical structure of EdUTP. (**c**) 5-TAMRA labeling on the denatured DNA strand by click reaction.

We designed a template-dependent nucleotide incorporation assay based on the use of biotin-labeled-primers and specific alkyne-modified dNTPs in an enzymatic reaction for dNTP quantitation. The alkyne-modified dNTP strand was immobilized on streptavidin beads, and subjected to a copper(I)-catalyzed alkyne-azide cycloaddition (CuAAC) “click” reaction. The azide-fluorophore probe was conjugated to the alkyne-dNTP-labeled strand for quantification by an ELISA reader. As an example, different amounts of dCTP were incubated with a reaction mixture containing a specific oligonucleotide template pre-annealed with a biotin-labeled primer, DNA polymerase, and an excess amount of 5-ethynyl-dUTP (EdUTP). The DNA polymerization reaction incorporates several molecules of EdUTP per molecule of dCTP incorporated. After termination of the reaction, streptavidin Sepharose was added to pull-down the oligonucleotides, which were then denatured with 0.1N NaOH and washed extensively to remove free EdUTP and template. Biotin-labeled strands with incorporated EdU on beads were conjugated with 5-TAMRA-azide via the click reaction, and fluorescence intensity on beads was measured to quantify the amount of dCTP incorporated (Fig. 1a). The chemical structure of EdUTP and click reaction are showed in Fig. 1b,c, respectively.

### Optimization of oligonucleotide templates for establishing standard curves

**Figure 2:**
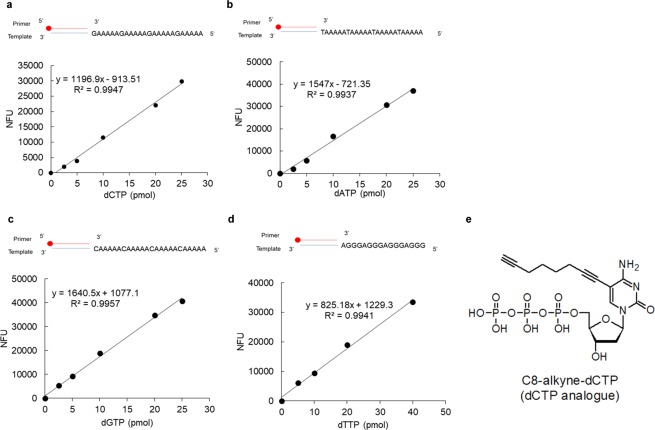
Standard curves for dNTP measurements. Templates as indicated were incubated with Zgene Taq polymerase in the presence of increasing amounts of (**a**) dCTP, (**b**) dATP and (**c**) dGTP together with EdUTP as described in Materials and Methods. (**d**) For dTTP measurement, templates were incubated with Vent (exo-) in the presence of known amounts of dTTP and C8-Alkyne-dCTP. The oligonucleotides pull-down using streptavidin Sepharose were labeled with 5-TAMRA-azide by click reaction. Fluorescence units of control reactions without dNTPs were subtracted from the values obtained in order to give normalized fluorescence units (NFU). (**e**) The chemical structure of C8-alkyne-dCTP.

Three different templates were designed for the click reaction to establish a standard curve for dCTP. Oligonucleotides of four repeats of dGdAdA (dG-dA_2_), dGdAdAdAdA (dG-dA_4_), and dGdAdAdAdAdA (dG-dA_5_) that shared a common sequence at 3′end were synthesized (Table S1). These oligomers were hybridized to the same biotin-5′-labeled primer, acting as templates for the DNA polymerization reaction in the presence of both dCTP (0–50 pmol) and an excess amount of EdUTP (1,000 pmol) in each reaction. Accordingly, two, four and five molecules of EdUTP per dCTP were incorporated when using the (dG-dA_2_)_4_, (dG-dA_4_)_4_, and (dG-dA_5_)_4_ templates, respectively. In this study, we chose Zgene Taq DNA polymerase, which lacks exonuclease activity and efficiently discriminates dNTPs from rNTPs^19^. The reaction was performed at 60 °C, followed by pull-down using streptavidin beads, denaturation and the click reaction. After measurement using an ELISA reader, the normalized fluorescence units (NFU) obtained were proportional to the amount of dCTP present in the reaction. The assay was linear up to 50 pmol of dCTP (Fig. S1A,B) for both (dG-dA_2_)_4_ and (dG-dA_4_)_4_ while poly(dG-dA_5_)_4_ exhibited linearity from 2.5 to 25 pmol (Fig. 2a). Calibration curves of the three templates gave a linearity coefficient r^2^ > 0.99. However, the slope of each calibrated standard curve revealed that the sensitivity of the (dG-dA_5_)_4_ template was higher than that of the poly(dG-dA_4_) and poly(dG-dA_2_) templates (Supplementary Fig. S1, Fig. 2a). We then used (dT-dA_5_)_4_ and poly(dC-dA_5_)_4_ templates to establish standard curves for dATP and dGTP, respectively. The calibration curves of dATP and dGTP showed similar linearity (r^2^ > 0.99) and sensitivity (Fig. 2b,c).

For the dTTP standard curve, EdUTP was replaced by C8-alkyne-dCTP (Fig. 2e). However, the (dA-dG_5_)_4_ template employed in the reaction using Zgene Taq polymerase gave limited incorporation of C8-alkyne-dCTP. It has been reported that Vent (exo-) polymerase shows better polymerization efficiency when using GC rich templates and presents low discrimination between canonical and modified nucleotides^20,21^. Thus poly(dA-dG_5_) and (dA-dG_3_) were tested as templates in reactions with Vent (exo-) polymerase in the presence of C8-alkyne-dCTP. The standard curve for dTTP using the (dA-dG_3_)_4_ oligomer exhibited good linearity (r^2^ > 0.99) and high sensitivity (Fig. 2d), while with the (dA-dG_5_)_4_ template, only poor incorporation was attained (data not shown).

Considering that (dA-dG_3_)_4_ oligomer has the potential to form G-quadruplex^22^, we also assessed the possible complication of G-quadruplex in the polymerase reaction by incubation of the template with Thioflavin T (ThT), which is a benzothiazole dye that gives fluorescence upon binding to G-quadruplex^23,24^. Like the human telomeric sequence, 22AG, the template (dA-dG_3_)_4_ did form G-quadruplex at 25 °C. However, at 60 °C, which used for the polymerization reaction, little G-quadruplex was detected for the template (Supplementary Fig. 2). Thus, the reaction temperature we used can avoid G-quadruplex formation.

### The competition effect of cellular nucleotides on quantitation

**Figure 3:**
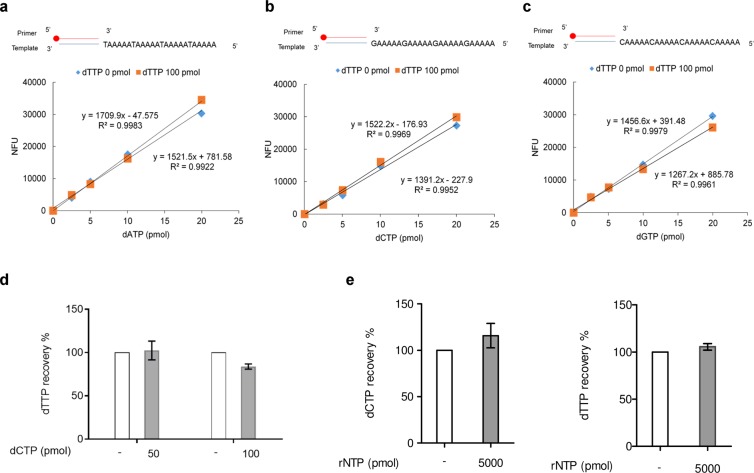
Effect of nucleotides on dNTP quantification by click assay. (**a**–**c**) Standard curves for (**a**) dATP, (**b**) dCTP, (**c**) dGTP were performed without- or with 100 pmol of dTTP and using Taq polymerase. 5-TAMRA-labeling of streptavidin Sepharose was determined by fluorescence quantitation. The red circle at the 5′ end of the primer indicates biotin. (**d**) Recovery of 20 pmol of dTTP analyzed by C8-alkyne-dCTP incorporation by Vent (exo-) polymerase in the presence of 50 pmol dCTP or 100 pmol dCTP. (**e**) Recovery of 10 pmol of dCTP and 20 pmol of dTTP were analyzed in the presence of rNTP mixtures (5000 pmol of each ATP, CTP, GTP and UTP). Bars represent mean ± SEM and assays were performed in triplicate.

According to existing data from several reports using LC/MS/MS methodology, the levels of dTTP and dCTP in 10^6^ cells range from 10 to 100 and 2 to 20 pmol, respectively^15,16^. Bearing in mind that dTTP may compete with EdUTP and dCTP with C8-alkyne-dCTP, we tested the effect of including 100 pmol of dTTP in the reactions when determining the standard curves for dATP, dCTP and dGTP. The results showed that the inclusion of 100 pmol of dTTP had little effect on the sensitivity and linearity of the resulting standard curves (Fig. 3a–c).

For dTTP quantification, the use of 1,000 pmol of C8-alkyne-dCTP for labeling may lead to potential competition between C8-alkyne-dCTP and dCTP during DNA polymerization, as described by Cahova *et al*.^25^. We therefore tested the effect of including 50 pmol or 100 pmol of dCTP in the dTTP quantification assay. The recovery of 20 pmol of dTTP was unaffected by the presence of 50 pmol of dCTP in the reaction (Fig. 3d). Hence, since the level of dCTP in 10^6^ cells is usually less than 20 pmol^15,19,26–29^, the interference of cellular levels of dCTP in the determination of dTTP should be negligible.

It has been shown that misincorporation of rNTPs in DNA polymerization is a potential source of interference in dNTP quantification^19^. Since the cellular ratio of rNTP/dNTP could be over 100, a mixture of four rNTPs (5,000 pmol each) was added to the reactions for dCTP and dTTP measurement. The recovery analysis revealed that a high level of rNTPs has no effect on dNTP quantitation (Fig. 3e). This is consistent with previous studies showing that Taq and Vent (exo-) polymerase discriminate very efficiently dNTPs from rNTPs^19,30^.

We further compared click and radioisotope methodology in the quantitation of the four dNTP pools in K562 and HEK-293T cells. Here, the radioisotope method using Zgene Taq polymerase was based on previously described procedures. Data obtained for the four dNTPs in K562 and HEK-293T cells by these two methods were similar (Table 1). Therefore, the click method can efficiently replace the radioisotopic assay when performing dNTP determinations in cell extracts.

**Table 1:** Comparison of cellular concentrations of dNTPs for K562 cells quantified by the click method and radioisotope method^15^.

	pmol/10^6^ cells				Ratio of (A + T)/(C + G)
	dATP	dTTP	dCTP	dGTP	
**K562**					
Click	10.9 ± 2.8	33.2 ± 4.5	6.9 ± 3.9	5.9 ± 4.0	3.4
Radioisotope	9.9 ± 1.5	27.6 ± 2.2	9.9 ± 3.3	3.1 ± 0.4	2.9
**HEK-293T**					
Click	17.1 ± 5.9	93.6 ± 19.8	10.1 ± 2.2	11.3 ± 2.8	5.2
Radioisotope	18.6 ± 9.8	92.5 ± 14.2	9.9 ± 3.0	13.0 ± 5.8	4.9

Each data point represents the mean ± SD determined from two independent isolations and analyzed at least in duplicate.

To know the extraction efficiency of methanol extraction in nucleotides recovery, we compared ATP levels in 2 different cell lines, HEK-293T and HCT116 cells, that before and after methanol extraction using CellTiter-Glo (Supplementary Fig. 3). Data showed that the extraction efficiency was 100%.

### The microplate assay for quantitating changes in dNTP pools

**Figure 4:**
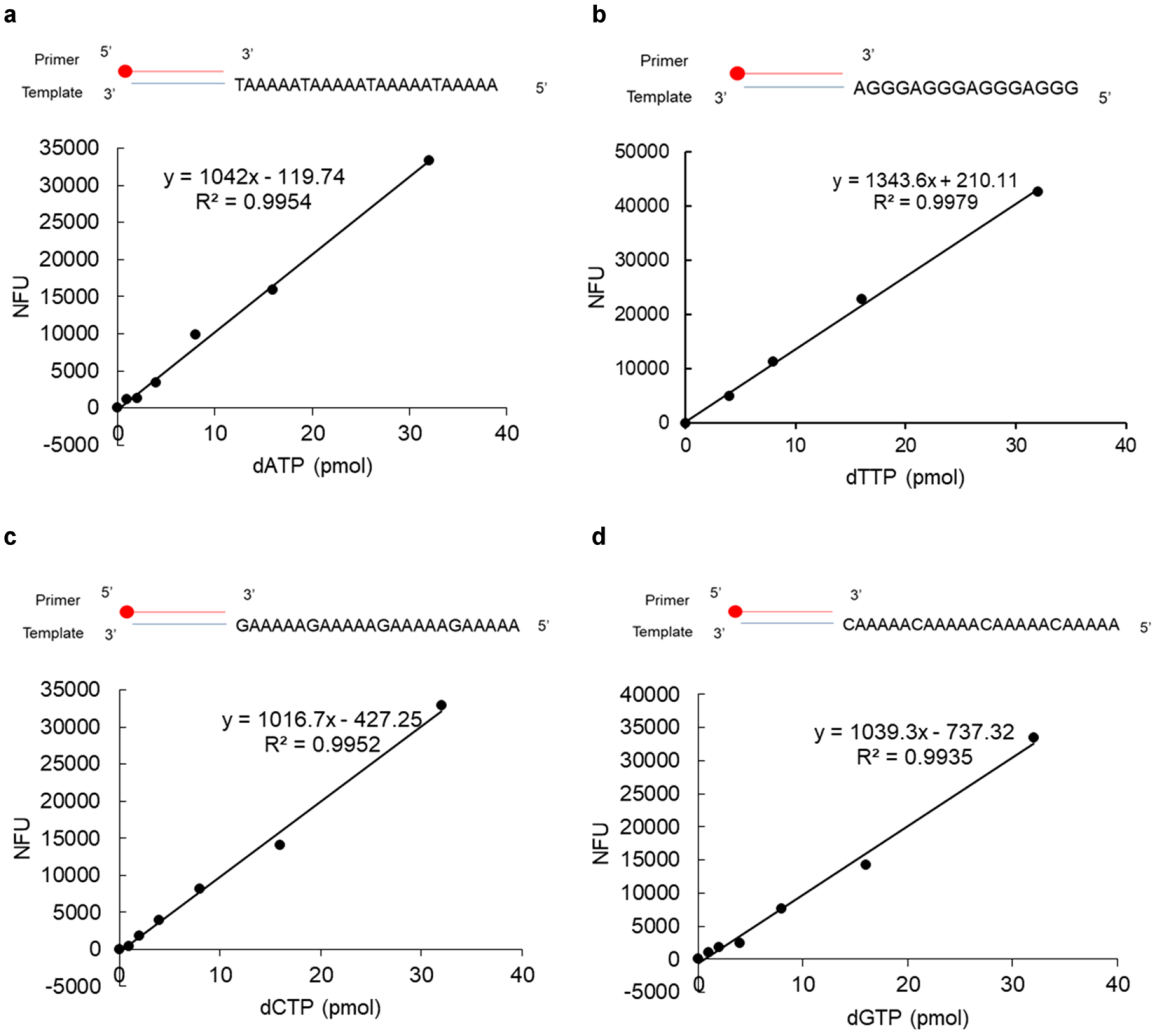
Standard curves for dNTP measurements in a microplate assay. The reactions, pull-downs, denaturing steps, 5-TAMRA-labeling and washings were performed in a 96-well microplate as described in Materials and Methods. The standard curves are shown for (**a**) dATP, (**b**) dTTP, (**c**) dCTP, and (**d**) dGTP. Fluorescence units of control reactions without dNTPs were subtracted from the values obtained in order to give normalized fluorescence units (NFU).

To improve the assay capacity, the principle of aforementioned method for each dNTP measurement was further adapted to a 96-well-microplate analysis using streptavidin magnetic Sepharose placed on a magnetic separator to perform all pull-down and washing steps in the same plate. For dATP, dCTP and dGTP assays, each reaction contained 2,000 pmol of EdUTP to avoid the potential competition of high dTTP pools in some particular cell lines. The reaction volumes and other parameters of both the primer-extension and click reactions are the same as described using streptavidin Sepharose in tubes, except the washing volumes were decreased to 200 μL for each sample. The linear range for dATP, dCTP, and dGTP was from 1 to 32 pmol (Fig. 4a,c,d). For dTTP, the linearity ranged from 4 pmol to 32 pmol (Fig. 4b). All standard curves exhibited r^2^ > 0.99.

### Determination of cellular dNTP pools in response to chemotherapeutic agents

**Figure 5:**
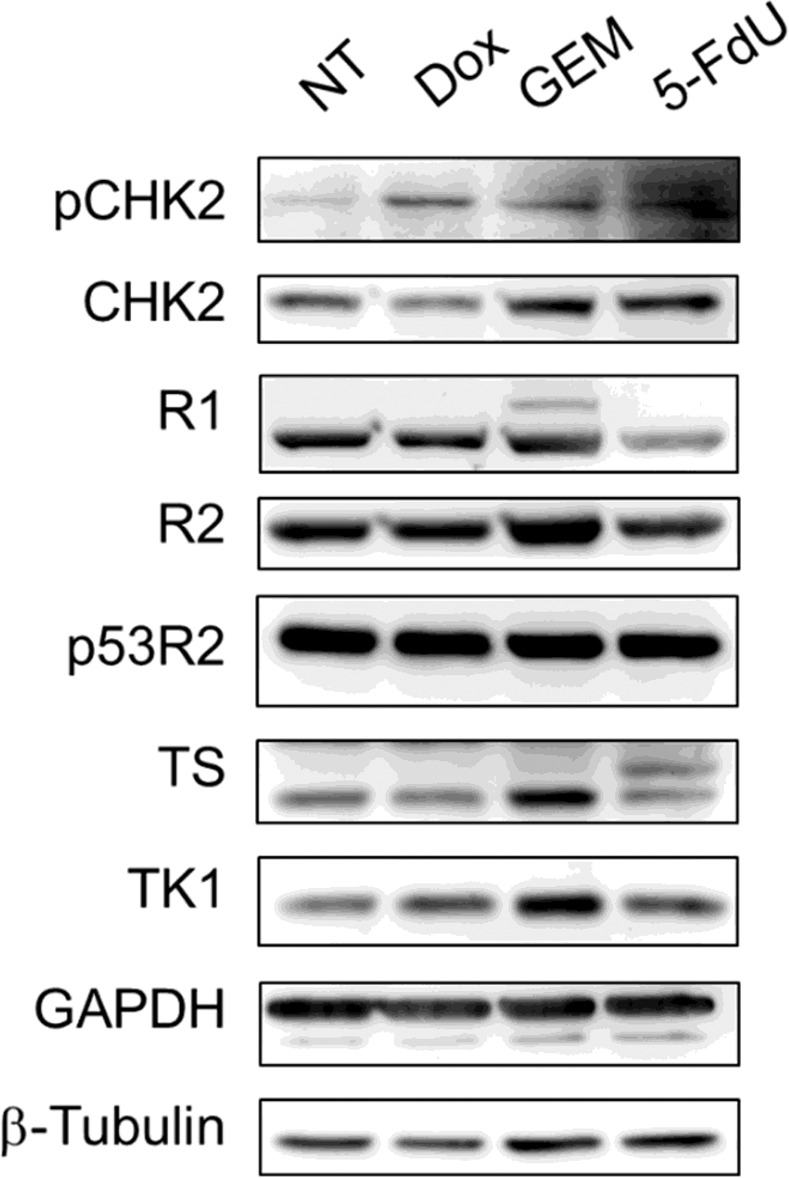
Western blot analysis of protein levels in response to different chemotherapeutic agents. HCT116 cells were treated with doxorubicin (Dox, 1 μM) for 1 hr, followed by recovery for 8 hr. In parallel, cells were treated with Gemcitabine (GEM, 1 μM) and 5-fluoro-deoxyuridine (5-FdU, 2 μM) for 8 hr and 6 hr, respectively. Cells were harvested for Western blot analysis.

We then used the microplate assay to measure dNTP levels in response to short-term treatment with different chemotherapeutic drugs including doxorubicin (Dox), gemcitabine (GEM) and 5-fluorodeoxyuridine (5-FdU), which are widely used in cancer treatment. Doxorubicin inhibits and stabilizes topoisomerase II, causing genotoxicity^31^. GEM inhibits ribonucleotide reductase (RNR) activity, blocking *de novo* synthesis of dNTPs^11^. 5-FdU is metabolically converted into 5-fluoro-deoxyuridine monophosphate (5-FdUMP), which covalently modifies and inhibits thymidylate synthase, consequently blocking *de novo* synthesis of dTTP^32–34^. A mismatch repair-deficient colon cancer cell line, HCT116, was used for the drug treatments. Cells were exposed to Dox (1 μM) for 1 hr and recovered in fresh medium for 8 hr. These conditions presumably allow for the modulation of dNTPs in response to the activation of DNA repair. Cells were also treated with GEM (1 μM) for 8 h or 5-FdU (2 μM) for 6 h. After treatment, cells were harvested for Western blot analysis and methanol extraction for the microplate assay. Western blot analysis revealed that all the treatments caused an activation of the DNA damage checkpoint response, as indicated by the increase in phospho-CHK2 (pThr68). We found that both the expression levels of subunits of R1, R2, p53R2, thymidylate synthase (TS) and thymidine kinase 1(TK1) and the four dNTP pools remained unchanged in HCT116 cells after recovery from Dox exposure (Table 2). GEM treatment for 8 h clearly results in the depletion of dATP and dGTP pools while dTTP levels were increased probably due to upregulation of both TS and TK1 (Fig. 5). As for 5-FdU treatment for 6 h, we found that 90% of the dTTP pool was depleted accompanied by a 75% and 55% reduction in dCTP and dGTP, respectively, with no obvious change in dATP levels. It was noted that levels of the R1 subunit of RNR diminished upon 5-FdU treatment (Fig. 5), which might contribute to the reduction of dCTP and dGTP (Table 2).

**Table 2:** Effect of three anti-cancer agents on the pools of four dNTPs in HCT116 cells.

HCT116				
Treatment	pmol/10^6^			
	control	Dox(1 μM)	GEM (1 μM)	5-FdU (2 μM)
	—	1 hr	8 hr	6 hr
Recovery	—	8 hr	—	—
dATP	2.6 ± 0.2	3.9 ± 0.8	Non detectable ↓(0)	1.9 ± 0.6
dTTP	7.6 ± 3.4	8.1 ± 2.1	14.1 ± 0.9 ↑ (1.9)	Non detectable ↓(0)
dCTP	1.2 ± 0.9	2.0 ± 1.6	0.9 ± 0.6	Non detectable ↓(0)
dGTP	7.1 ± 0.8	5.8 ± 1.9	1.8 ± 1.1 ↓ (0.3)	3.2 ± 0.3 ↓ (0.45)

Each data point represents the mean ± SD determined from two independent experiments in duplicate. Numbers in parentheses indicate fold changes relative to the control. ↑ and ↓ indicate the significant increase and decrease relative to the control. Dox, doxorubicin. GEM, gemcitabine. 5-FdU, 5-fluorodeoxyuridine

## Discussion

The levels of cellular dNTP pools provide important information indicating metabolic status for DNA synthesis. Information regarding alterations in dNTP pools in response to treatment with anti-cancer agents in cancer and immune cells is of paramount interest when developing precision medicine taking into account the context of molecular networks. Therefore, the availability of a convenient and versatile method for quantification of dNTPs is greatly sought after. In this study, we designed a method which combines enzymatic and click reactions to quantitate four dNTP pools and further developed a 96-well microplate assay to increase the assay capacity. Using this microplate assay, we compared the effect of treatment with three different anti-cancer agents on four dNTP pools in HCT116 cells. Our data revealed that gemcitabine treated cells exhibit reduced dGTP and increased dTTP levels, together with the upregulation of TK1 and TS. On the other hand, 5-FdU treatment not only results in a depletion of the dTTP pool, as expected, but also caused a marked reduction in the dCTP and dGTP pools. Since HCT116 is a colon cancer cell line, the severe reduction in three dNTPs by 5-FdU treatment is in agreement with 5-FU as the mainstay in adjuvant chemotherapy of colon cancer. Thus, the microplate assay developed in this study would provide an important tool for evaluating the contribution of dNTP alterations to disease development and the effect of drug action on therapy.

The current method used one common biotin-labeled primer annealed with four different templates, each specific for dATP, dTTP, dGTP and dCTP. The amount of EdUTP or C8-alkyne-dCTP incorporation was correlated with the amount of dATP/dCTP/dGTP or dTTP respectively. The method was designed for determination of each pool in 10^6^ cells. Previous analyses of cellular dNTP levels in human cells have provided values in the range of 4–30 pmol for dATP, 10–100 pmol for dTTP, 2–20 pmol dCTP, and 2–20 pmol for dGTP in 10^6^ cells^15,19,26–29^. The assay here provided is therefore suitable for dCTP and dGTP quantitation whereas extract dilution may be required for cell lines that contain high levels of dATP or dTTP in order to ensure measurements within linearity.

Adapting the assay to a microplate format with magnetic beads has made the assay convenient for handling washing steps, thus increasing sample throughput. One major concern during optimization was that high cellular levels of dTTP might result in an underestimation of dATP, dCTP and dGTP due to competition with EdUTP. The current protocol contains 1,000 pmol of EdUTP in each reaction, which presumably is sufficient to overcome the competition effect derived from high levels of dTTP. In the 96-well microplate assay, the amount of EdUTP in each assay was increased to 2,000 pmol and the linear range for each dNTP was expanded to 1–32 pmol. Overall, we propose the use of click reactions as a replacement for conventional methodology using radioisotopes in dNTP quantitation. In addition, requirements for a scintillation counter and institutional regulations including purchases, disposal, storage and a specialized operating place can be all avoided with this new technology.

## Methods

### Chemicals, drugs and reagents

Oligonucleotides were synthesized by Genomics Bioscience and Technology Co., Ltd (Taiwan, ROC). C8-alkyne-dCTP and 5-ethynyl-dUTP were purchased from Jena Bioscience or Baseclick. Tetramethylrhodamine azide isomer 5 (5-TAMRA-azide) was from Lumiprobe. Taq polymerase was from ZGene Biotech Inc. Vent (exo-) DNA polymerase was purchased from New England Biolabs. CuSO4, Tris (3-hydroxypropyltriazolylmethyl) amine (THPTA), phosphatase inhibitor, NaF, Na_3_VO_3_, thioflavin T (ThT) and sodium ascorbate were from Sigma^19^. ^3^H-dATP (30.7 Ci/mmol) was purchased from Moravek Biochemical, and 1, 2 methyl-^3^H-dTTP (79.3 Ci/mmol) from PerkinElmer. dATP, dGTP, dCTP, dTTP were from sigma. Streptavidin Sepharose and streptavidin magnetic Sepharose were from GE Healthcare. CellTiter-Glo was from Promega.

### Cell growth and extract preparation for dNTP quantitation

Human chronic myeloid leukemia cell line K562 cells were maintained in RPMI1640 (Gibco) containing 10% heated inactivated fetal bovine serum (HIFBS; BI, biological industries) and penicillin/streptomycin. HEK-293T cells were maintained in DMEM (Gibco) with 10% HIFBS and penicillin/streptomycin. HCT116 cells were maintained in McCoy’s 5A(Gibco) with 10% FBS and penicillin/streptomycin. For drug treatment, HCT116 cells were plated in 10-cm tissue culture dishes at 2 × 10^6^ to grow for 48 h. Cells were then treated with vehicle, doxorubicin (Dox), gemcitabine (GEM), or 5-fluorodeoxyuridine (5-FdU). After treatment, cells were rinsed twice with cold PBS to remove the remaining medium. The adherent cells were then detached by trypsin-EDTA and were washed twice with 10 mL cold PBS and then counted before centrifuged. Cells (10^6^) were extracted with 1 mL of 60% ice-cold methanol and placed at 95 °C for 5 min. The extracts were centrifuged (12,000× g, 30 min) to remove cell debris. The supernatants were then evaporated by SpeedVac (Thermo) and the resultant pellet was stored at −80 °C until use. The dry residue was dissolved in water and used for assays. For each dNTP quantitation, 20 μL of extract from 10^6^ cells was added to the 30 μL reaction buffer as described in dNTP standard curves.

To determine the extraction efficiency, an aliquot of 5000 cells after cell counting was stored at −80 °C until used. The remaining cells were subjected to methanol extraction as described above. Cell extracts from 5000 cells were transferred to a white 96-well, together with the aliquot of 5000 cells as indicated previously. CellTiter Glo (Promega) was then added to each sample for ATP measurement. The luminescence units were detected by Tecan Spark 10 M.

### Template-dependent primer extension reaction of dNTP standard curves

Biotin-labeled primer and the designated template were annealed at a final concentration of 10 μM in a buffer (300 mM NaCl, 50 mM Tris-HCl, 10 mM MgCl_2_, 100 μg/ml BSA, pH 7.9). After heating at 95 °C for 5 min, the solution was cooled to 25 °C at the slow ramp rate (0.1 °C/min). For dATP, dCTP and dGTP determination, a primer extension reaction in a final volume of 50 μl contained 0.6 μM template-primer, 20 μM EdUTP in 1× Zgene Taq buffer, 20 μL of serial dilution of dNTP standard, and the reaction was initiated by adding 2 units of Zgene Taq polymerase. For dTTP determination, 20 μL of serial dilution of dTTP standard and 20 μM C8-alkyne-dCTP were used in 1× ThermoPol Reaction Buffer Pack with 0.5 U Vent (exo-) DNA Polymerase. All reactions were performed in tubes and were incubated at 60 °C for 40 min. Oligonucleotides in this study are listed in Table S1.

### Pull-down of oligonucleotides and copper (I) catalyzed azide-alkyne cycloaddition (CuAAC) reaction

Streptavidin Sepharose slurry in PBS was added to each reaction tube, followed by addition of NaOH at the final concentration of 0.1 N. The Sepharose beads were washed once with a 500 μL buffer (10 mM Tris, 1 mM EDTA, 100 mM NaCl and 0.1% SDS), followed by 2 × 500 μL of ddH_2_O. After washing, CuAAC reaction mixture (100 μL) containing 10 μM azide (5-Carboxytetramethylrhodamine azide), 5 mM sodium ascorbate and Cu (II) solution was added to the beads in each tube. The solution was prepared freshly by mixing CuSO_4_ and Tris(3-hydroxypropyltriazolylmethyl)amine (THPTA) at final concentrations of 0.5 mM and 2.5 mM, respectively. The reaction mixture was incubated at room temperature for 1 h, followed by washing once with a 500 μL buffer (10 mM Tris-HCl, pH 7.5, 1 mM EDTA, 100 mM NaCl and 0.1% SDS) and twice with 500 μL of ddH_2_O. All beads were transferred to a black microplate (Greiner). The fluorescence was measured by Tecan Spark 10 M. Excitation wavelength λ^ex^ and emission wavelength λ^em^ for 5-TAMRA was 510 nm and 560 nm, respectively. Standard curves were generated every time when quantification of cellular dNTP pools.

### Modification for microplate assay

The conditions for primer extension were as aforementioned except for a 2-fold increase in the concentration of EdUTP to avoid the potential competition of high dTTP pools in some cell extracts. All reactions in a volume of 50 μL were performed in a black microplate with a clear bottom and incubated at 60 °C for 40 min. To pull-down biotin-labeled DNA, 50 μL of streptavidin magnetic Sepharose (GE Healthcare) slurry in PBS was added to each reaction. After denaturation by 0.1N NaOH, the microplate was placed on a (96-Well) EpiMag HT magnetic separator to carry out the washing steps to remove the template strand by using a multichannel pipette: twice with a 200 μL buffer (10 mM Tris-HCl, pH 7.5, 1 mM EDTA, and 0.1% SDS) and four times with 200 μL of ddH_2_O. After washing, 100 μL of the CuAAC reaction (10 μM 5-TAMRA-azide, 5 mM sodium ascorbate and premixed Cu (II)-THPTA solution) was added to magnetic Sepharose beads, followed by washing steps as described above. Fluorescence in 96-well plate was measured by Tecan Spark 10 M. Excitation wavelength λ^ex^ and emission wavelength λ^em^ for 5-TAMRA was 510 nm and 560 nm, respectively. Standard curves were generated every time in duplicate when quantification of cellular dNTP pools

### Quantification of cellular dNTPs using radioisotope-labeling assay

The general conditions of the enzymatic-based assay were as previously described^19,26^. Briefly, 0.25–8 pmol of dNTP standard were added to the reaction mixture containing 40 mM Tris-HCl, pH 7.4, 10 mM MgCl_2_, 5 mM dithiothreitol (DTT), 0.25 μM oligonucleotide (Table S1), 1.5 μg RNase A, 0.25 μM [^3^H]dATP (500–1000 cpm/pmol) for dCTP, dTTP, dGTP, or 0.25 μM [^3^H]dTTP for dATP quantification, and Zgene Taq polymerase in a final volume of 100 μL. After incubation at 48 °C for 60 min, reaction mixtures were spotted on Zeta-Probe Blotting Membranes (Bio-Rad). After drying, membranes were washed three times for 5 min, first in 5% Na_2_HPO4, once in distilled water and finally in 95% ethanol. Radioactivity on membranes was measured by scintillation counting.

### Western blot analysis

Cells were lysed with RIPA buffer containing 100 mM Tris, pH 7.4, 150 mM NaCl, 0.5 mM EDTA, 1 mM EDTA, 0.5% NP-40, 0.1% SDS, 0.2% deoxycholate, 25 mM β-glycerophosphate, 10 mM phenylmethylsulfonyl fluoride, 2 mM Na_3_VO_3_, 10 mM NaF, 1× protease inhibitor cocktail 2, and 1× phosphatase inhibitor. After sonication, cell lysates containing 20 μg of protein were separated by 12% SDS-PAGE and transferred to polyvinylidene fluoride membranes. After blocking with 5% heated non-fat milk, membranes were incubated with primary antibody overnight at 4 °C. Antibodies used are: R1(Santa Cruz), R2(Santa Cruz), p53R2(Santa Cruz), CHK2 (Millipore), pCHK2 (Thr68; Cell signaling), thymidylate synthase (TS, ZYMED), Glyceraldehyde 3-phosphate dehydrogenase (GAPDH; GeneTex), thymidine kinase 1(TK1)^35^, and β-Tubulin (Sigma). Membranes were then treated with horseradish peroxidase-conjugated secondary antibodies (Santa Cruz) for 1 hr, followed by ECL detection according to the manufacturer’s instructions (Millipore).

### Statistical analysis

Data are presented as the mean ± SD or mean ± SEM. Statistical comparison of means was performed using a Mann Whitney U test.

## Supplementary information

Supplementary Information.
