# Supplementary material for: Quantitation of deoxynucleoside triphosphates by click reactions
Source: Sci Rep. 2020 Jan 17;10:611. doi: 10.1038/s41598-020-57463-3 (PMC6969045; doi:10.1038/s41598-020-57463-3)
Supplement: Supplementary file 1 — Supplementary Information. [file 41598_2020_57463_MOESM1_ESM.pdf]

Table S1. Oligonucleotides in this research

Oligonucleotide	Sequence
poly(dT-dA_5) [a]	5'-AAAAA**T**AAAAA**T**AAAAA**T**AAAAA**T**GGCGGTGGAGGCGG 3'
poly(dC-dA_5)[a]	5'-AAAAA**C**AAAAA**C**AAAAA**C**AAAAA**C**GGCGGTGGAGGCGG-3'
poly(dG-dA_5)[a]	5'-AAAAA**G**AAAAA**G**AAAAA**G**AAAAA**G**CGGCGGTGGAGGCGG-3'
poly(dG-dA_4)[a]	5'-AAAA**G**AAAA**G**AAAA**G**AAAA**G**CGGCGGTGGAGGCGG-3'
Poly(dG-dA_2)[a]	5'-AA**G**AA**G**AA**G**AA**G**CGGCGGTGGAGGCGG-3'
poly(dA-dG_3)[a]	5'-GGG**A**GGG**A**GGG**A**GGG**A**GGCGGTGGAGGCGG-3'
Biotin dNTP primer 1	5'-biotin-CCGCCTCCACCGCC-3'
Biotin dNTP primer 2	5'-biotin-CCGCCTCCACCGCCG-3'
dATP template [b]	5'-AAA**T**AAA**T**AAA**T**AA**T**AAA**T**GGCGGTGGAGGCGG -3'
dGTP template [b]	5'-AAA**C**AAA**C**AAA**C**AAA**C**AAA**C**GGCGGTGGAGGCGG -3'
dCTP template [b]	5'-AAA**G**AAA**G**AAA**G**AAA**G**AAA**G**GGCGGTGGAGGCGG -3'
dTTP template [b]	5' TT**A**TT**A**TT**A**TT**A**TT**A**TT**A**GGCGGTGGAGGCGG 3'
Primer[b]	5'-CCGCCTCCACCGCC-3'

[a] Bases in bold represents the site where the limiting dNTP will base pair

[b] The sequences of templates and primers were described by Sherman(23).

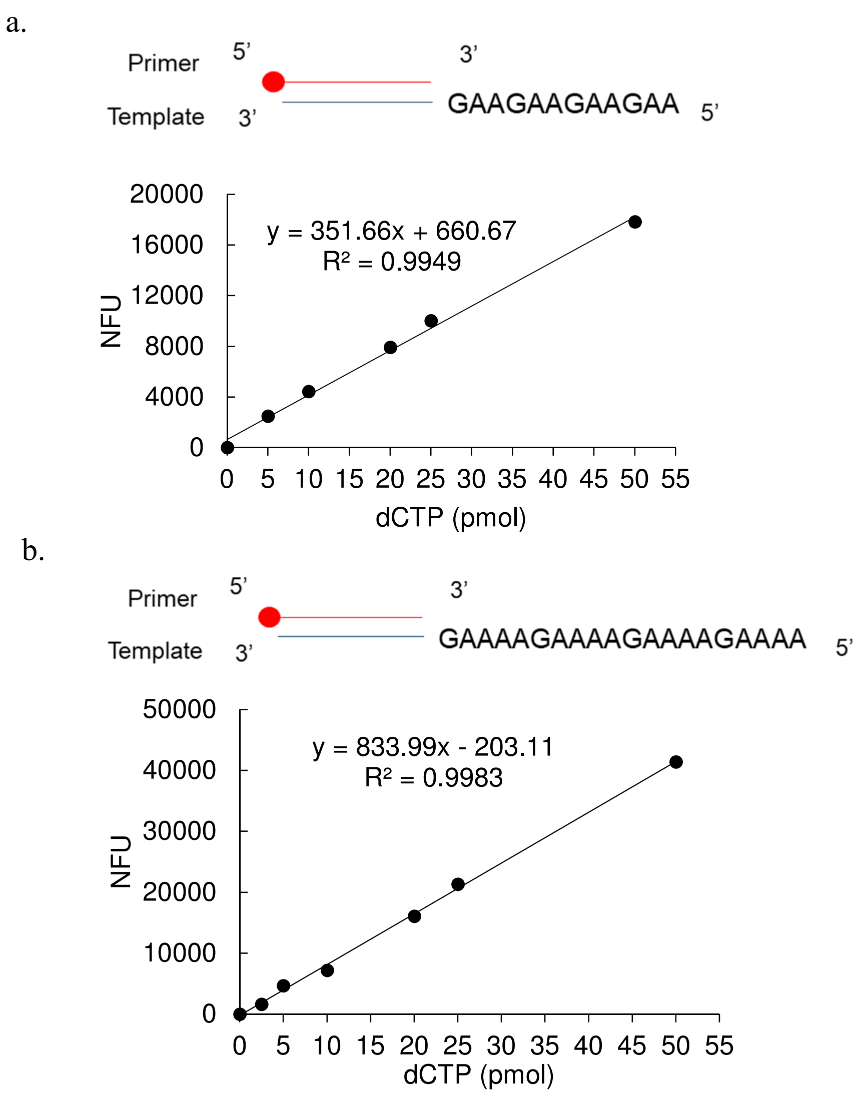

Supplementary Figure 1. Standard curves for dCTP measurement using different templates. The specific template was annealed with the biotin-labeled primer and incubated with EdUTP, the indicated amount of dCTP and with Zgene Taq as described in Materials and Methods. Standard curves (a) using poly(dG-dA2) and (b) poly(dG-dA4) as templates. Fluorescence of the control reaction without dCTP was subtracted from the values obtained in order to give normalized fluorescence units (NFU). Calibration curves for all templates showed $r^2 > 0.99$. The red circle at the 5' end of the primer indicates biotin.

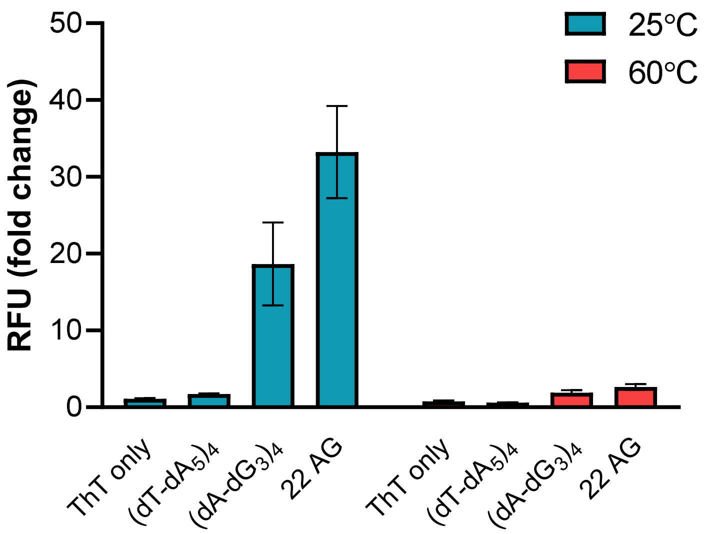

Supplementary Figure 2. The assessment of G-quadruplex formation in the reaction using $(dA\text{-}dG_3)_4$ template. Thioflavin T (ThT) at the final concentration of 1 μM) was added to 1X ThermolPol reaction buffer containing DNA (2.5 μM) as indicated at room temperature 25℃ or 60℃ for 10 min prior to fluorescence measurement. The fold change in fluorescent units relative to the that control reaction without DNA was shown. $(dT\text{-}dA_5)_4$: Template for dATP quantitation. $(dA\text{-}dG_3)_4$: Template for dTTP quantitation. 22 AG: a human telomeric DNA sequence, 5'-AGGGTTAGGGTTAGGGTTAGGG-3', as a positive control. Excitation wavelength λ^{ex} and emission wavelength λ^{em} was 430 nm and 490 nm, respectively. Bars represent mean ± SEM in triplicate.

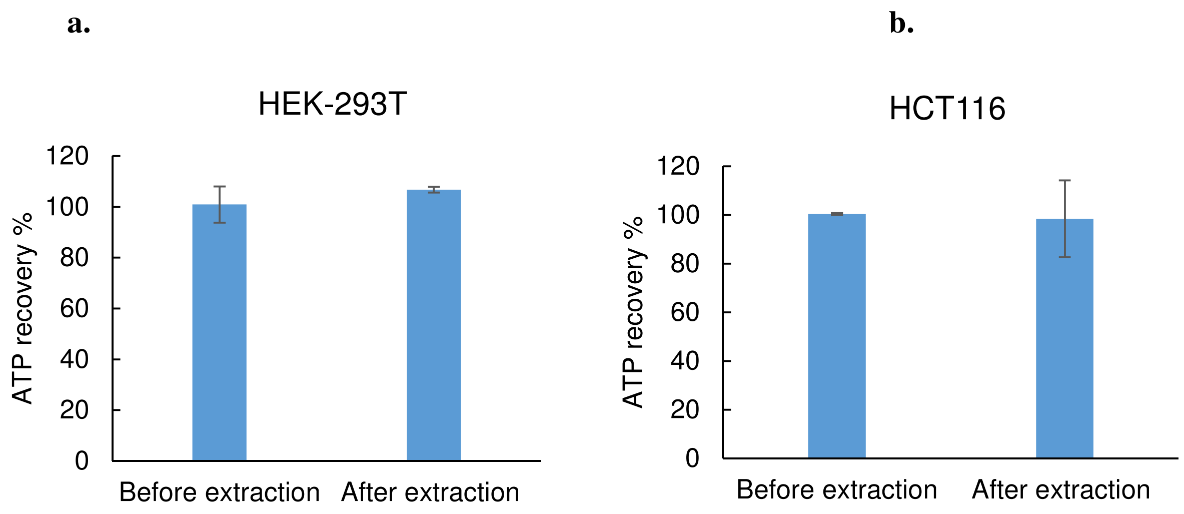

Supplementary Figure 3. Determination of ATP recovery from methanol extraction. The level of ATP in 5,000 cells before and after methanol extraction was determined by luciferase assay. Samples were suspended in 15 µL ddH_2O and mixed with 15 µL of CellTiter-Glo®. Luminescence unit was recorded after 10 minutes using Tecan Spark 10M. Bars represent mean ± SEM in triplicate. (a) HEK-293T, and (b) HCT116 cells.

Supplementary Figure 4

Figure 5 in manuscript

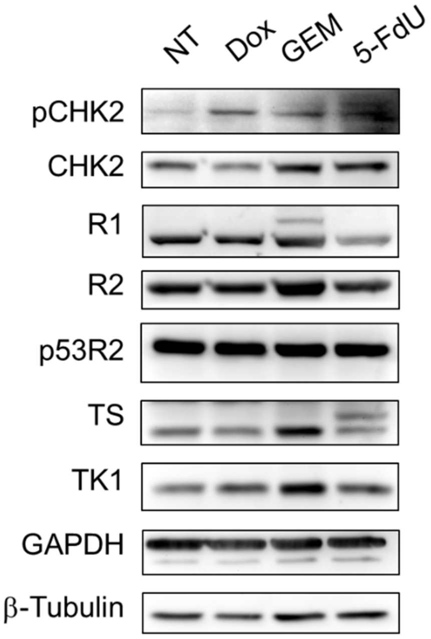

Raw Data:

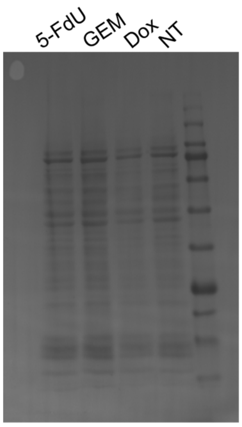

Blot 1:
R1
CHK2
R2
GAPDH
TK1

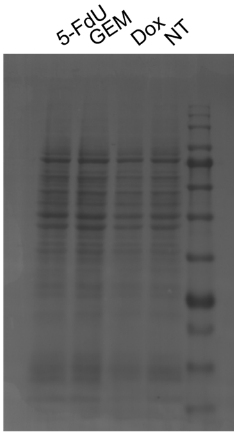

Blot 2:
pCHK2
β-Tubulin
p53R2
TS

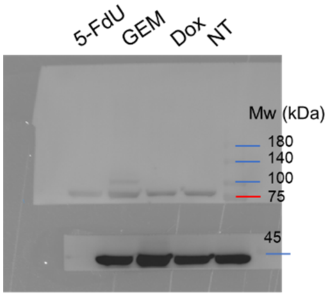

Western blot: R1 (top) and R2 (bottom)

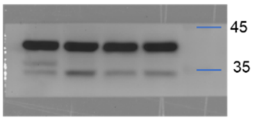

Western blot: p53R2 (top) and reprobed for TS (bottom)

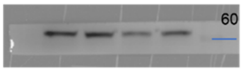

Western blot: CHK2

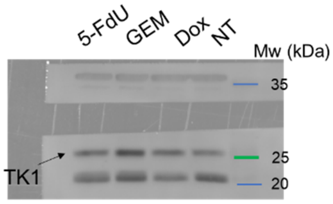

Western blot: GAPDH (top) and TK1 (bottom)

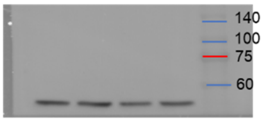

Western blot: β-tubulin

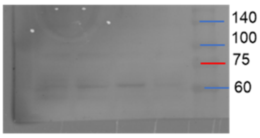

Western blot: pCHK2
